# Inhibitory effect of sodium butyrate on colorectal cancer cells and construction of the related molecular network

**DOI:** 10.1186/s12885-021-07845-1

**Published:** 2021-02-06

**Authors:** Yang Xi, Zhuang Jing, Wu Wei, Zhang Chun, Qi Quan, Zhou Qing, Xu Jiamin, Han Shuwen

**Affiliations:** 1 Department of Oncology, Huzhou Central Hospital, Affiliated Central Hospital Huzhou University, No.1558, Sanhuan North Road, Wuxing District, Huzhou, 313000 Zhejiang Province China; 2 Graduate School of Nursing, Huzhou university, No. 1 Bachelor Road, Huzhou, 313000 Zhejiang Province China; 3 Department of Gastroenterology, Huzhou Central Hospital, Affiliated Central Hospital Huzhou University, No.1558, Sanhuan North Road, Wuxing District, Huzhou, 313000 Zhejiang Province China; 4 Department of Infectious Disease, Huzhou Central Hospital, Affiliated Central Hospital Huzhou University, No.1558, Sanhuan North Road, Wuxing District, Huzhou, 313000 Zhejiang Province China; 5 Department of Nursing, Huzhou Central Hospital, Affiliated Central Hospital Huzhou University, No.1558, Sanhuan North Road, Wuxing District, Huzhou, 313000 Zhejiang Province China

**Keywords:** Sodium butyrate; colorectal cancer; molecular network; apoptosis; ceRNA

## Abstract

**Background:**

Sodium butyrate (NaB) is produced through the fermentation of dietary fiber that is not absorbed and digested by the small intestine.

**Purpose:**

Here, we aimed to investigate the effects of NaB on the proliferation, invasion, and metastasis of CRC cells and their potential underlying molecular mechanism(s).

**Methods:**

The cell counting kit-8 (CCK-8) assay and EdU assay were used to detect cell proliferation ability, flow cytometry was used to investigate the induction of apoptosis and cell cycle progression, and the scratch-wound healing and transwell assays were used to evaluate cell migration and invasion, respectively. The human CRC genome information for tissues and CRC cells treated with NaB obtained from the NCBI GEO database was reannotated and used for differential RNA analysis. Functional and pathway enrichment analyses were performed for differentially expressed lncRNAs and mRNAs. A protein-protein interaction (PPI) network for the hub genes was constructed using the Cytoscape software. Targeted miRNAs were predicted based on the lnCeDB database, and a ceRNA network was constructed using the Cytoscape software. The Kaplan-Meier method was used to analyze patient prognosis using the clinical information and exon-seq data for CRC obtained from the Broad Institute’s GDAC Firehose platform.

**Results:**

NaB decreased the proliferation ability of CRC cells in a dose- and time-dependent manner. The number of apoptotic CRC cells increased with the increase in NaB concentrations, and NaB induced a G1 phase block in CRC cells. Moreover, NaB suppressed the migratory and invasive capabilities of CRC cells. There were 666 differentially expressed mRNAs and 30 differentially expressed lncRNAs involved in the CRC inhibition by NaB. The PPI network and ceRNA network were constructed based on the differentially expressed mRNAs and lncRNAs. Three differentially expressed mRNAs, including HMGA2, LOXL2, and ST7, were significantly correlated with the prognosis of CRC.

**Conclusion:**

NaB induces the apoptosis and inhibition of CRC cell proliferation, invasion, and metastasis by modulating complex molecular networks. RNA prediction and molecular network construction need to be the focus of further research in this direction.

## Background

Colorectal cancer (CRC), a common malignant tumor, is one of the main causes of cancer-related deaths [1]. The rapid development of diagnostic methods, multidisciplinary diagnosis and treatment models, small-molecular targeted drugs, and immunotherapy drugs in recent years has significantly improved the diagnosis and treatment of CRC [2–5]. However, the prognosis of CRC patients needs to be improved further due to the unclear pathogenesis of CRC and lack of effective therapeutic drugs. Moreover, CRC treatment also levies a heavy economic burden on the patients [6]. There is still a lot to be done to ensure effective and inexpensive drugs for CRC.

Sodium butyrate (NaB) is a short-chain fatty acid produced by intestinal microflora during the anaerobic fermentation of dietary fiber [7]. It is readily available and inexpensive. As a component of intestinal microecological environment, its short-term effects on the human body are not obvious [8]. NaB can maintain the stability of the gut environment by regulating various cellular functions, including proliferation, differentiation, apoptosis, and intestinal epithelial permeability [7, 9, 10]. By regulating immune cells, including regulatory T cells, dendritic cells, natural killer cells, and macrophages, NaB inhibits the expression of HDAC [11], increases the acetylation of regulatory proteins, and regulates various molecular signaling pathways [12], thereby inhibiting the proliferation of malignant tumor cells and reducing the risk of cancer [13].

We confirmed the inhibitory effect of NaB on CRC cells in the present study. The molecular mechanism of NaB on CRC cells is complex, and there are inconclusive and inconsistent reports regarding this. Compiling the latest gene database information and by analyzing the genomic data of CRC cell lines after NaB intervention, a molecular network of the effect of NaB on CRC cells was constructed. In addition, the analysis of differentially expressed RNA related to CRC prognosis will provide support for further clinical application of NaB in CRC.

## Methods

### Cell culture

Human CRC cell lines SW480, LOVO, HCT116, and HCT8 were obtained from the Cell Bank of Type Culture Collection of Chinese Academy of Sciences (Shanghai, China). RPMI-1640 (Gibco; Thermo Fisher Scientific, Inc., Waltham, MA, USA) supplemented with 10% heat-inactivated fetal bovine serum (FBS; Gibco, Thermo Fisher Scientific, Inc.) was used as the cell culture medium. These CRC cell lines were incubated in a humidified incubator (Sanyo XD-101; Sanyo Electric Co., Ltd., Osaka, Japan) with 5% CO_2_ at 37 °C.

### CCK-8 assay

We seeded the exponentially growing CRC cell lines (5000 cells/100 μl) into 96-well plates, pre-incubated them for 24 h, added NaB at concentrations ranging from 0 to 32 mmol/L (10 μl), and then incubated them for 24, 48 and 72 h. Following this, we added 10 μl of CCK-8 solution (KGA317, Nanjing KeyGen Biotech Co., Ltd., Nanjing, China) into the 96-well plates and incubated them for 3 h. A microplate reader (ELx800; BioTek Instruments, Inc., Winooski, VT, USA) was used to measure the absorbance at 450 nm. Physiological saline was used as blank, and untreated CRC cells were used as the control. The inhibition rate (IR) was calculated as follows: IR = [1 - (A450 values of the experimental group – A450 values of the blank group) /(A450 values of the control group – A450 values of the blank group)] × 100%.

### Analysis of cell apoptosis

CRC cells (5 × 10^5^ cells/well) were treated with NaB at a concentration of 0, 2.5, 5, 10 mmol/L for 48 h at 37 °C and seeded in 6-well plates. Following this, they were washed twice with 0.1 mmol/L phosphate-buffered saline (PBS), mixed with 5 μl annexin V fluorescein isothiocyanate (FITC; KGA105; KeyGen Biotech Co., Ltd.) and propidium iodide (PI; KGA511, KeyGen Biotech Co., Ltd), incubated for 15 min at room temperature in the dark, and then analyzed using FACS flow cytometer (Becton.Dickinson, Franklin Lakes, NJ, USA) with an argon laser (488 nm). The cell number in each quadrant was calculated by the internal software system.

### Cell cycle analysis

We cultured CRC cells along with HCT116 cell lines and HCT8 cell lines in 6-well plates (3.0 × 10^5^ cells/well) with medium for 24 h, treated them with NaB (5 mmol/L) for 48 h, then washed, collected, and fixed the cells using 70% ethanol and stored them at 4 °C overnight. Following this, Tris-HCl buffer (pH 7.4) containing 1% RNase A (cat. no KGA511; KeyGen Biotech Co., Ltd) was added to the cells and they were stained using PI (5 mg/ml) in each well. Flow cytometry (FACSCalibur; Becton-Dickinson) and cell cycle analysis software (FlowJo, version 7.6.5; KeyGen Biotech Co., Ltd.) were used to analyze the data. The experiment was repeated three times for each sample.

### Scratch-wound assay

CRC cells were cultured in 12-well plates, which then formed mono-layer cells; after this, a line was scratched using a sterile 20-ul pipette tip, and the cultured monolayer cells were washed three times and then treated with NaB (5 mmol/L) for 24 h. The width of the scratch area was used to estimate the migration capacity.

### Transwell invasion assay

CRC cells were collected and cultured with 200 μL serum-free medium in the upper chambers of Matrigel-coated transwell plates (2 × 10^4^ cells per well) with a pore size of 8 μm; cell culture medium was added to the lower chamber. After incubation for 48 h at 37 °C and 5% CO_2_, the cells were fixed with a 4% formaldehyde solution for 30 min and then stained with a 0.4% crystal violet solution for 10 min at room temperature. Cells that invaded into the bottom of the transwell membranes in each chamber were randomly selected and photographed with a microscope. The invasion capacity of untreated HCT8 and HCT116 cells and those treated with NaB (5 mmol/L) was assessed by counting the number of cells in 5 microscope fields (200 folds).

### Statistical analysis

SPSS software version 18.0 (SPSS, Inc., Chicago, IL, USA) was used for statistical analyses. Differences between measurement date for the groups were assessed using independent sample T tests. Cell counting data were assessed using the chi-square test. *P* < 0.05 was considered to indicate a statistically significant difference.

### Molecular network construction

#### Data processing and re-annotation

The data set No. GSE54127 (species: *Homo sapiens*) from the NCBI GEO [14] (Gene Expression Omnibus, GEO, http://www.ncbi.nlm.nih.gov/geo/) database includes human genome information from 6 HCT116 cell samples and 6 HCT116 cells treated with NaB. Data were obtained using Agilent-026652 Whole Human Genome Microarray 4x44K v2 (Probe Name version) platform. The original chip probe signals were processed using data standardization, including background correction, normalization, and the determination of expression using the RMA (robust multi - array business) method in the R [15] affy package (Version 1.52.0, http://www.bioconductor.org/packages/2.9/bioc/html/affy.html). The human reference genome (GRCh38) from the GENCODE database [16] (https://www.gencodegenes.org/releases/current.html) was compared to the above probe sequences by using seqmap software [17]. Probes with unique maps were retained, and the corresponding genes for each probe were identified based on chromosome position and positive- and negative-chain information according to the human gene annotation file (Release 25) provided by GENCODE. Annotation information for the corresponding probe “protein_coding” probe was as mRNA, while that for the corresponding “antisense”, “sense_intronic”, “lincRNA”, “sense_overlapping” or “processed_transcript” probes was as lncRNA. Finally, probes that did not match gene symbols were removed. The mean values for different probes were taken as the final mRNA/lncRNA expression values.

#### Differential RNA analysis

The classical Bayesian method provided via the limma package [18] (Version 3.10.3, http://www.bioconductor.org/packages/2.9/bioc/html/limma.html) was used to analyze the differential mRNA and lncRNA between the experimental group (treated with NaB) and control group (HCT116). The Benjamini & Hochberg method [19] was used to perform multiple inspection and correction for the corresponding *p* value and logFC value. The *p* value < 0.05 after the correction and |logFC| > 2 (4-fold change) was as the difference expression threshold.

#### Functional and pathway enrichment analysis

The R package clusterProfiler [20] (version: 3.8.1, http://bioconductor.org/packages/release/bioc/html/clusterProfiler.html) was used to perform the Gene Ontology BP (biological process) [21] and KEGG [22] pathway enrichment analysis with regard to differentially expressed mRNA. Pearson correlation coefficients were obtained for differentially expressed mRNAs and lncRNAs. Relationships with r > 0.9 and p value< 0.05 were screened for subsequent ceRNA network construction. The mRNA was as potential target gene of lncRNA. The KEGG pathway enrichment analysis for the mRNA corresponding to the lncRNA was carried out using the clusterProfiler package in R, and lncRNA function was indirectly predicted via the enrichment analysis results. An adjusted *P* value < 0.05 was considered to indicate significant enrichment after “BH” correction.

#### Protein interaction (PPI) network construction

The interaction between the proteins encoded by the differentially expressed genes obtained from the above analysis was analyzed based on the STRING database (Version:10.0, http://www.string-db.org/) [23]. The species was set to *Homo sapiens* and the PPI score was set to 0.9 (highest confidence). The Cytoscape software [24] was used to construct the network basing on the PPI pairs. The CytoNCA plug-in [25] (Version 2.1.6, http://apps.cytoscape.org/apps/cytonca) was used to analyze the topological properties of the node network. The parameter was set to without weight and the results included Degree Centrality (DC), Betweenness Centrality (BC), and Closeness Centrality (CC). The important nodes (hub proteins) involved in protein interaction in the PPI network were determined by ranking the topological properties of each node.

#### MiRNA prediction and ceRNA network construction

The lnCeDB [26] database was used to predict the targeted miRNA corresponding to the differentially expressed lncRNA based on the lncRNA-mRNA pairs identified I the above analyses. Six databases—miRWalk, miRanda, miRDB, PITA, RNA22, and Targetscan—and miRWalk2.0 software [27] (http://zmf.umm.uni-heidelberg.de/apps/zmf/mirwalk2/) were used to predict the targeted miRNA corresponding to the differentially expressed mRNAs. The lncRNA-miRNA-mRNA pairs were obtained by screening the mRNA-miRNA pairs and lncRNA-miRNA pairs regulated by the same miRNA. The Cytoscape software was used to construct the ceRNA network via analysis of the correlation of the lncRNA-miRNA-mRNA pairs (Correlation coefficient > 0.9). CytoNCA was used to analyze the degree of connectivity of each node in the ceRNA network.

#### Survival analysis of differential mRNA and lncRNA

Clinical information and exon-seq data of colorectal cancer (COADREAD) from the Broad Institute’s GDAC Firehose platform (http://gdac.broadinstitute.org/) were used for the survival analysis of differential mRNAs and lncRNAs. Chromosome position annotation data for lncRNA and protein-coding RNA in Gencode database were compared with the expression profile data of exon-seq. The starting and ending positions of an exon contained lncRNA or mRNA in the annotated database, and was consistency with the positive and negative chains. Finally, 369 cases of cancer with survival information were obtained by comparison of samples,

Clinical information, including overall survival (OS) and OS status, was included. The lncRNAs and mRNAs identified in the above ceRNA network were selected as candidate genes. The expression value of these candidate genes was screened using TCGA data, and divided into high expression and low expression groups based on the median expression value. The log-rank statistical test, with a threshold of *p* < 0.05, was conducted. The relationship between candidate genes and patient prognosis was analyzed, and the K-M survival curve was plotted.

### qPCR analysis

Human colorectal cancer cell lines HCT116 and HCT8 from the Chinese Academy of Sciences were selected. HCT116 and HCT8 cells were treated with 5 mmol/L NaB for 48 h as the experimental group, and not treated as the control group. Our research group cultured cell lines in RPMI-1640 (Gibco; Thermo Fisher Scientifc, Inc., Waltham, MA, USA), which mixed with 10%FBS and 1%P/S, and incubated them in a humidified incubator (Sanyo XD-101; Sanyo Electric Co., Ltd., Osaka, Japan) at 37 °C and 5% CO_2_.Using TRIzol reagent (Invitrogen; Yi Sheng Biotechnology Scientifc, China) extracted total RNA from those cells according to its instruction to reverse transcription and amplify PCR. Using the FTC-3000 real-time fluorescence quantitative PCR system (Funglyn Biotech, Inc., Ontario, Canada) performed PCR reactions. The reaction conditions are as follows. First, performe for 3 min at 50 °C, and then Increase temperature to 95 °C for 3 min. After 10s, cool to 60 °C for 30 s. The reaction is repeated over 40 cycles. The experiment was repeated three times. Researcher measured the relative expression of HMGA2, LOXL2 and ST7 by the 2^-△△Ct^ method. The GAPDH was regarded as a reference gene. Information on primers was shown in Table 1.

**Table 1:** Primers of genes used for qRT-PCR

Gene symbol	Primer (5′ to 3′)
GAPDH-hF	GAAGGTGAAGGTCGGAGTC
GAPDH-hR	GAAGGTGAAGGTCGGAGTC
HMGA2-F	CGAAAGGTGCTGGGCAGCTCCGG
HMGA2-R	CCATTTCCTAGGTCTGCCTCTTG
LOXL2-F	GGGTGGAGGTGTACTATGATGG
LOXL2-R	CTTGCCGTAGGAGGAGCTG
ST7-F	CGCGGATCCCCTCTGTGTGTGTGTGTGTAAC
ST7-R	CCGGAATTCGCATTCCTGGGCAGGTCGGT

### EdU assay

The EdU assay was performed to detect cell proliferation by using the KeyFluor488 Click-it EdU imaging kit (Jiangsu Kaiji Biotechnology Co., LTD., China, KGA331–100). The SW480 cells treated with 5 mmol/L NaB for 48 h, which were the experimental group, oppositely untreated cells were the control group. After cell digestion and counting, cell suspension with a concentration of 1 × 104 /mL was prepared. After adding 200 μL cell suspension (2 × 103 cells/well) to each well of 96-well cell culture plates, the plates were placed in an incubator at 37 °C with 5% CO2 for 24 h.After repeated cleaning, incubation, and decolorization shaker incubation, a high-content cell imaging system (Top Biotek Co, Ltd., USA, ImagExpress Micro HCS) was used for detection.

## Results

### NaB inhibits the proliferation of CRC cells

**Fig. 1:**
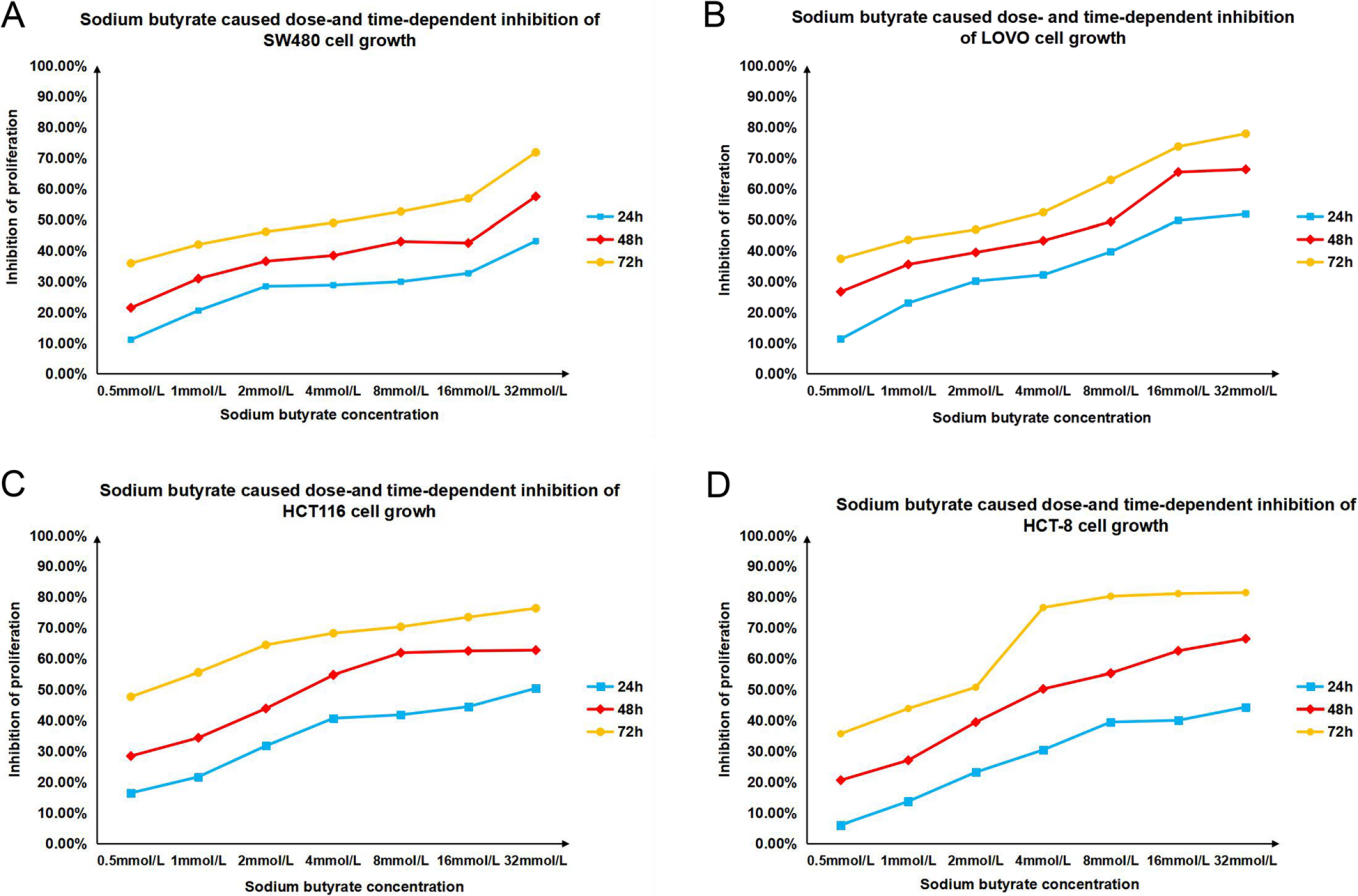
Sodium butyrate (NaB) inhibited the proliferation of colorectal CRC cell lines. The Cell counting kit-8 (CCK-8) assay was used to determine the effects of NaB on the proliferation of CRC cell lines. Panels **a**, **b**, **c**, and **d** represent the inhibition rate of NaB on SW480, LOVO, HCT116, and HCT8 cell lines, respectively

The effects of NaB on the proliferation of CRC cells, including the SW480, LOVO, HCT116, and HCT8 cell lines, were evaluated. As shown in Fig. 1, NaB had inhibitory effects on CRC cell proliferation. NaB showed cytotoxic effects against all the four cell lines. For SW480 cells, the IC50 (half maximal inhibitory concentration) values at 24 h, 48 h, and 72 h post treatment were 88.24 mmol/L, 19.31 mmol/L, and 3.67 mmol/L, respectively. For LOVO cells, the IC50 values at 24 h, 48 h, and 72 h post treatment were 20.18 mmol/L, 5.75 mmol/L, and 2.11 mmol/L, respectively. For HCT116 cells, the IC50 values at 24 h, 48 h and 72 h post treatment were 22.79 mmol/L, 4.10 mmol/L, and 0.45 mmol/L, respectively. For HCT8 cells, the IC50 values at 24 h, 48 h and 72 h post treatment were 31.63 mmol/L, 5.78 mmol/L, and 1.28 mmol/L, respectively. These results indicate that NaB decreased the viability of CRC cells in a dose- and time-dependent manner.

### NaB induces apoptosis of CRC cells in vitro

**Fig. 2:**
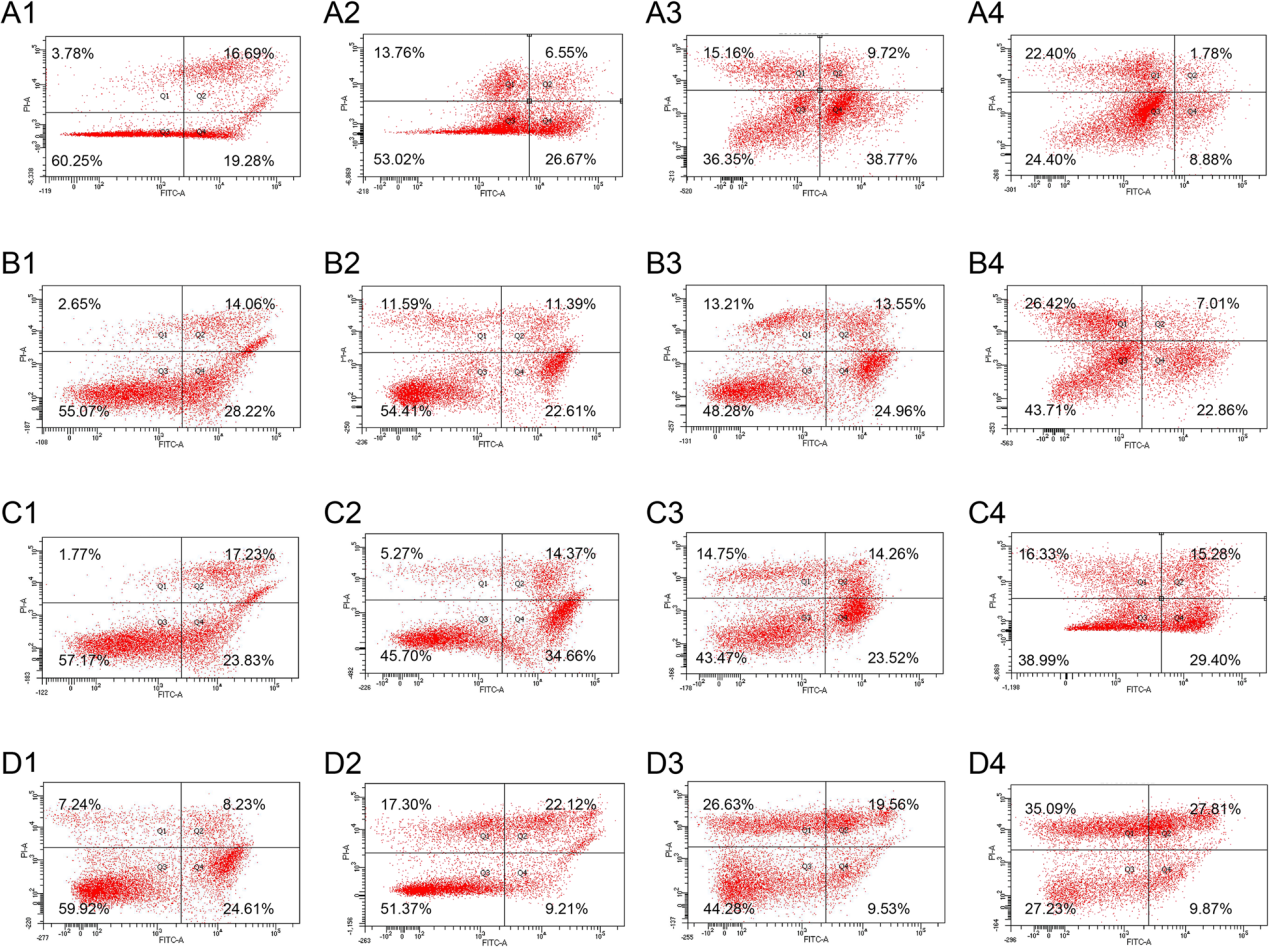
NaB induces apoptosis of CRC cells. NaB was determined by flow cytometric analysis of FITC-Annexin V and propidium iodide (PI) staining. The number of cells in each quadrant in this figure are representative of (Q1) necrosis, (Q2) late apoptosis, (Q3) live cells, and (Q4) early apoptosis. The panels A1–4, panels B1–4, panels C1–4, and panels D1–4 represent the SW480, LOVO, HCT116, and HCT8 cell lines. The panels A1, B1, C1, and D1 represent the cells untreated with NaB (control group). The panels A2, B2, C2, and D2 represent the cells treated with 2.5 mmol/L NaB. The panels A3, B3, C3, and D3 represent the cells treated with 5 mmol/L NaB. The panels A4, B4, C4, and D4 represent the cells treated with 10 mmol/L NaB

To evaluate apoptosis of CRC cells, the SW480, LOVO, HCT116 and HCT8 cell lines were treated with NaB at 0, 2.5, 5, and 10 mmol/L for 48 h. The quadrants Q1, Q2, Q3, and Q4 represent the number of dead cells, the number of late apoptotic cells, the number of live cells, and the number of early apoptotic cells, respectively. As shown in Fig. 2, the results revealed that the proportion of apoptotic CRC cells increased with the increase in the concentrations of NaB.

### NaB inhibits the cell cycle of CRC cells in vitro

**Fig. 3:**
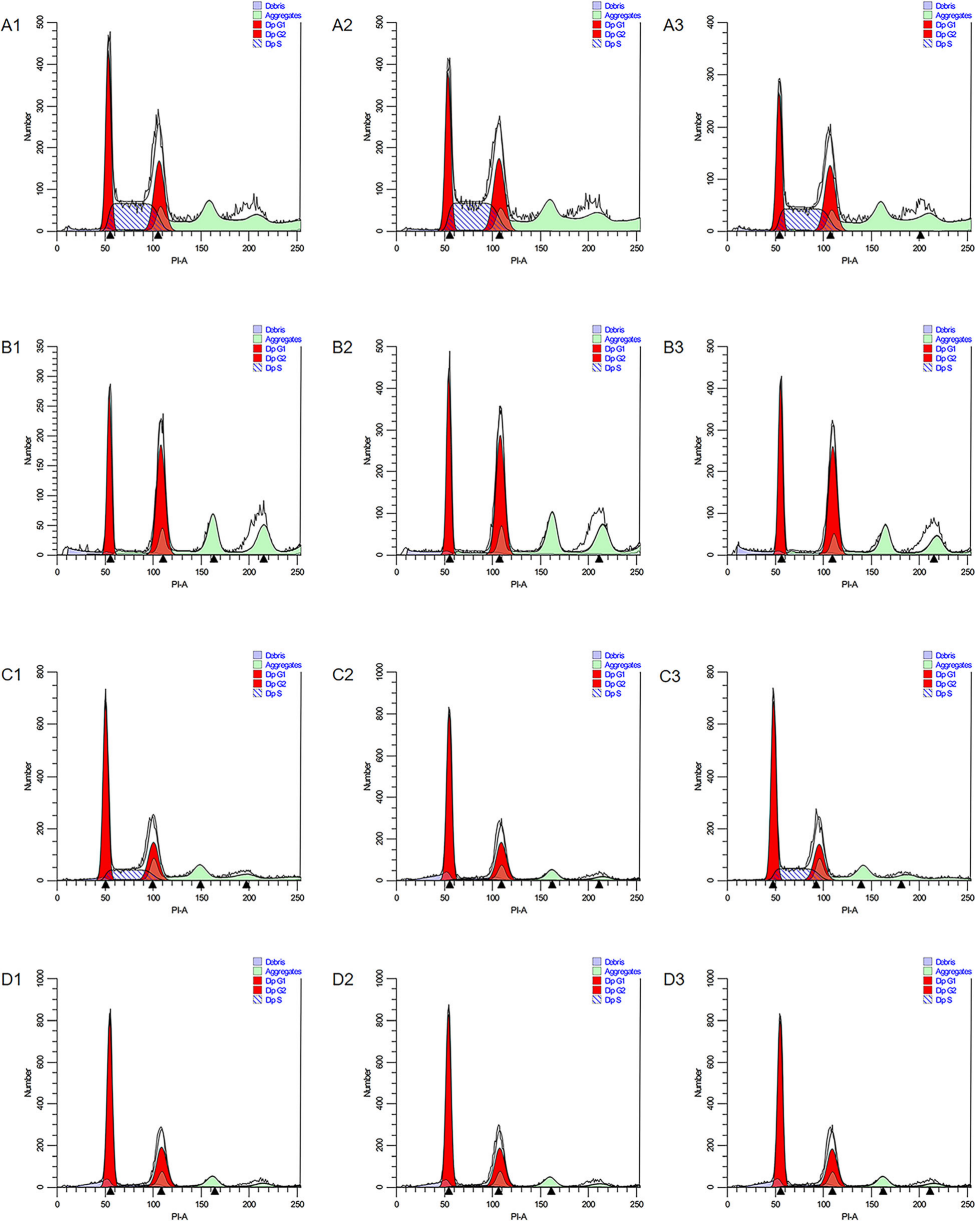
NaB inhibits the cell cycle phase of CRC cells. Cell cycle analysis of CRC cells treated with NaB (5 mmol/L) for 48 h was performed. Each test was performed in triplicate. The cell cycle was analyzed using flow cytometry. The panels A1–3, B1–3, C1–3, and D1–3 represent HCT116 cells, HCT116 cells treated with NaB, HCT8 cells, and HCT8 cells treated with NaB, respectively

Cell cycle analysis of CRC cells treated with NaB (5 mmol/L) for 48 h was performed. As shown in Fig. 3, the percentage of the sub-G1 population in HCT116 and HCT8 cells treated with NaB was higher than that in the control group (untreated CRC cells). Specific percentages and statistical analysis are shown in Table 2. The results suggested that NaB induced the arrest of the cell cycle at the G1 phase in CRC cells.

**Table 2:** NaB inhibits the cell cycle of CRC cells

Groups	Cell cycle(^−^*X ,* %)			Groups	Cell cycle(^−^*X,* %)		
HCT116	G1	S	G2	HCT-8	G1	S	G2
Control goup	32.05%	40.06%	27.89%	Control goup	54.29%	23.55%	22.16%
Sodium butyrate	43.72%	5.10%	51.18%	Sodium butyrate	67.38%	1.96%	30.66%
X^2^	3.06	35.13	11.07		3.54	21.40	2.08
p	0.08	< 0.001	0.001		0.06	< 0.001	0.15

Cell cycle analysis of CRC cells treated with sodium butyrate (5 mmol/l) for 48 h was performed. The table showed the specific percentages and statistical analysis of the cell cycle in HCT-116 and HCT-8 cells treated with sodium butyrate. The values in the table are the mean values measured by three parallel samples. The chi-square test was used to compare the rates between the NaB group and the control group

### NaB inhibits the migratory and invasive capabilities of CRC cells

**Fig. 4:**
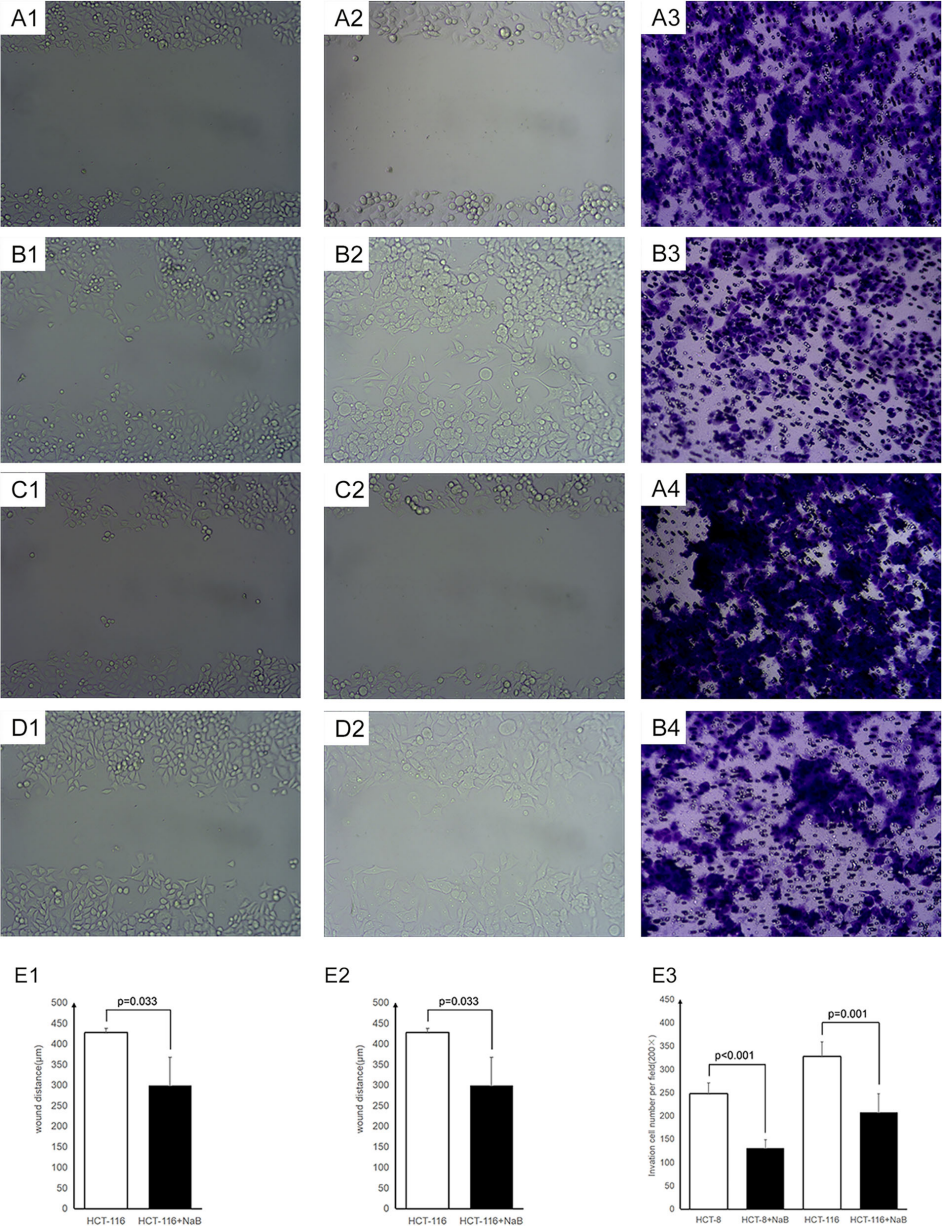
NaB inhibits the migration and invasion capacity of CRC cells. The wound healing assay and transwell invasion assay were used to evaluate the migration and invasion capacity, respectively, of the HCT8 and HCT116 cells. The wound distances of HCT8 cells untreated with NaB at 0 h (A1), HCT116 cells untreated with NaB at 0 h (A2), HCT8 cells untreated with NaB at 24 h (B1), HCT116 cells untreated with NaB at 24 h (B2), HCT8 cells treated with NaB at 0 h (C1), HCT116 cells treated with NaB at 0 h (C2), HCT8 cells treated with NaB at 24 h (D1), and HCT116 cells treated with NaB at 24 h (D2) were photographed. The statistical analysis of the wound distance of HCT8 cells and HCT116 cells is shown in panels E1 and E2, respectively (Student’s t test). The invading HCT8 cells untreated with NaB (A3), HCT8 cells treated with 5 mmol/L NaB (B3), HCT116 cells untreated with NaB (A4), and HCT116 cells treated with 5 mmol/L NaB (B4) were photographed under a microscope; the statistical analysis is shown in panel E3

Scratch-wound assays and Transwell assays were used to detect the cell migration and invasion of CRC cells, respectively, upon treatment with NaB. As shown in Fig. 4, the number of invasive HCT116 cells and HCT8 cells treated with NaB was significantly lower than that in the control group. Moreover, the scratch distance of HCT116 cells and HCT8 cells treated with NaB was significantly greater than that in the control group. The results suggested that NaB suppressed the migratory and invasive capabilities of CRC cells.

### Differentially expressed RNAs induced by NaB in colorectal cancer

**Fig. 5:**
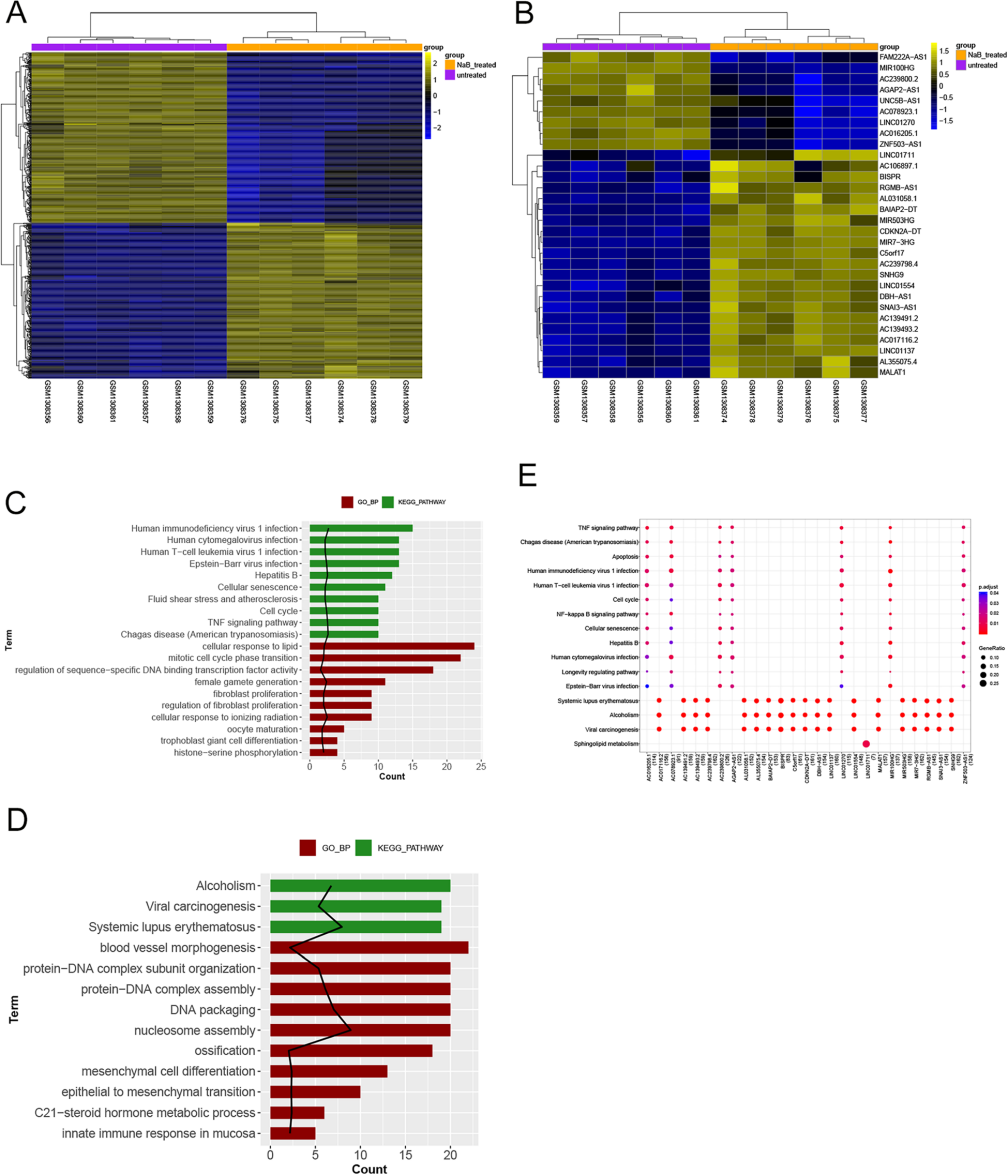
Differentially expressed RNAs induced by NaB in colorectal cancer and functional enrichment pathways. The heatmaps of panels **a** and **b** show the differentially expressed mRNAs and differentially expressed lncRNAs, respectively. The panels **c** and **d** show the pathway analysis results of down-regulated and up-regulated mRNAs (p.adjust ranked top10), respectively. Red color represents GO BP and green color represents KEGG pathway. The bar length indicates the number of genes enriched and the black line indicates -log10 (p.adjust). The panel E shows the results of lncRNA pathway analysis. The color range from blue to red indicates the decrease in *p* value, and the bubble size indicates the proportion of enriched genes (the number of genes involved in a certain term accounts for the number of input genes)

The gene sequences of six HCT116 cells samples and six HCT116 cells treated with NaB obtained from the NCBI GEO database were reannotated and analyzed. A total of 15,826 mRNA and 1967 lncRNA were annotated. According to the thresholds that were set in the method, the differentially expressed mRNA and lncRNA between the HCT116 cells treated with NaB and not treated with NaB were analyzed using the limma package. The panels A and B in Fig. 5 showed the differentially expressed mRNAs and differentially expressed lncRNAs, respectively. There were 666 differentially expressed mRNAs, including 318 upregulated mRNA and 348 downregulated mRNA, and 30 differentially expressed lncRNAs, including 21 upregulated lncRNA and 9 downregulated lncRNA, involved in NaB-mediated inhibition of CRC.

GO biological processes (BP) functional enrichment analysis and KEGG pathway enrichment analysis were conducted for the differentially expressed mRNAs. The up-regulated mRNAs significantly enriched 28 GO BPs and 3 KEGG pathways, whereas the down-regulated mRNAs significantly enriched 20 BPs and 29 KEGG pathways. Panels C and D of Fig. 5 show the pathway analysis results for the down-regulated and up-regulated mRNAs (p.adjust ranked top10), respectively. To predict the function of the corresponding lncRNAs, the KEGG pathway enrichment analysis was conducted using the mRNAs as the target genes for the corresponding lncRNAs based on the mRNA-lncRNA pairs that are known to be coexpressed. Panel E of Fig. 5 shows the 16 statistically significant KEGG pathways enriched by the differentially expressed lncRNAs. Comprehensive analysis of the results of functional enrichment revealed that pathways, including the TNF signaling pathway, human immunodeficiency virus 1 infection, human T-cell leukemia virus 1 infection, Epstein-Barr virus infection, cell cycle-related pathways, and apoptosis may participate in the inhibitory effect of NaB on CRC.

### Protein-protein interactions induced by NaB in CRC

**Fig. 6:**
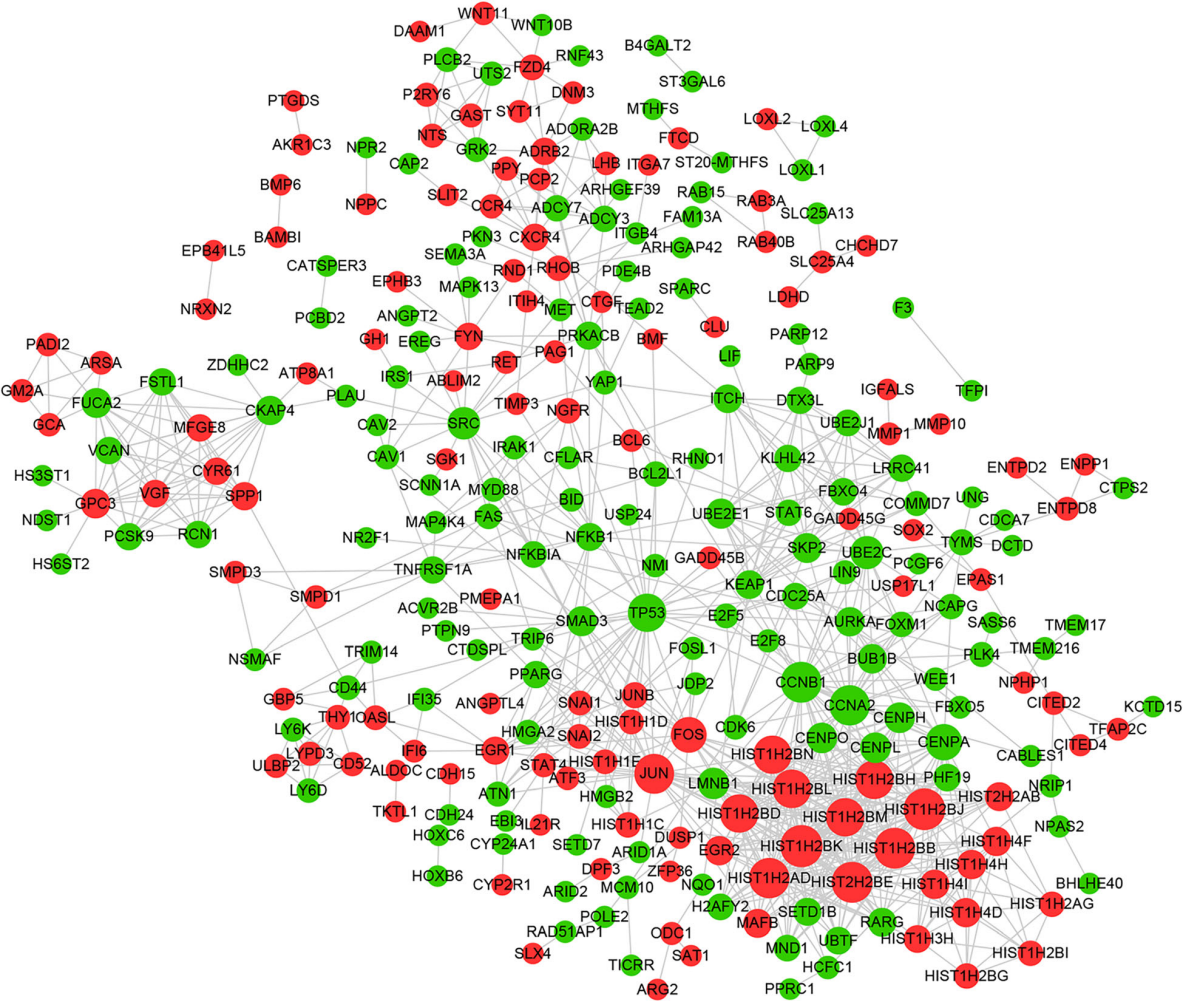
Protein-protein interactions network. The interaction between 666 differentially expressed mRNA coding proteins was predicted by using STRING database. The PPI network was constructed based on the protein-protein interactions (PPI) pairs. Red color represents up-regulated proteins, green color represents down-regulated proteins, and gray line represents protein interaction

The interaction between 666 differentially expressed mRNAs encoding proteins was predicted using the STRING database. A total of 841 protein interaction pairs were obtained, including 280 protein-coding mRNAs. Based on the protein-protein interaction (PPI) pairs, a PPI network was constructed using the Cytoscape software (Fig. 6). A node network topology analysis was conducted on the PPI network. The degree centrality (DC), betweenness centrality (BC), and closeness centrality (CC) of the top 15 nodes are shown in Table 3 four proteins, CCNA2, CCNB1, JUN, and TP53, were identified in the DC, BC, and CC of each of the top 15 nodes. These four proteins might be hub proteins in the network.

**Table 3:**
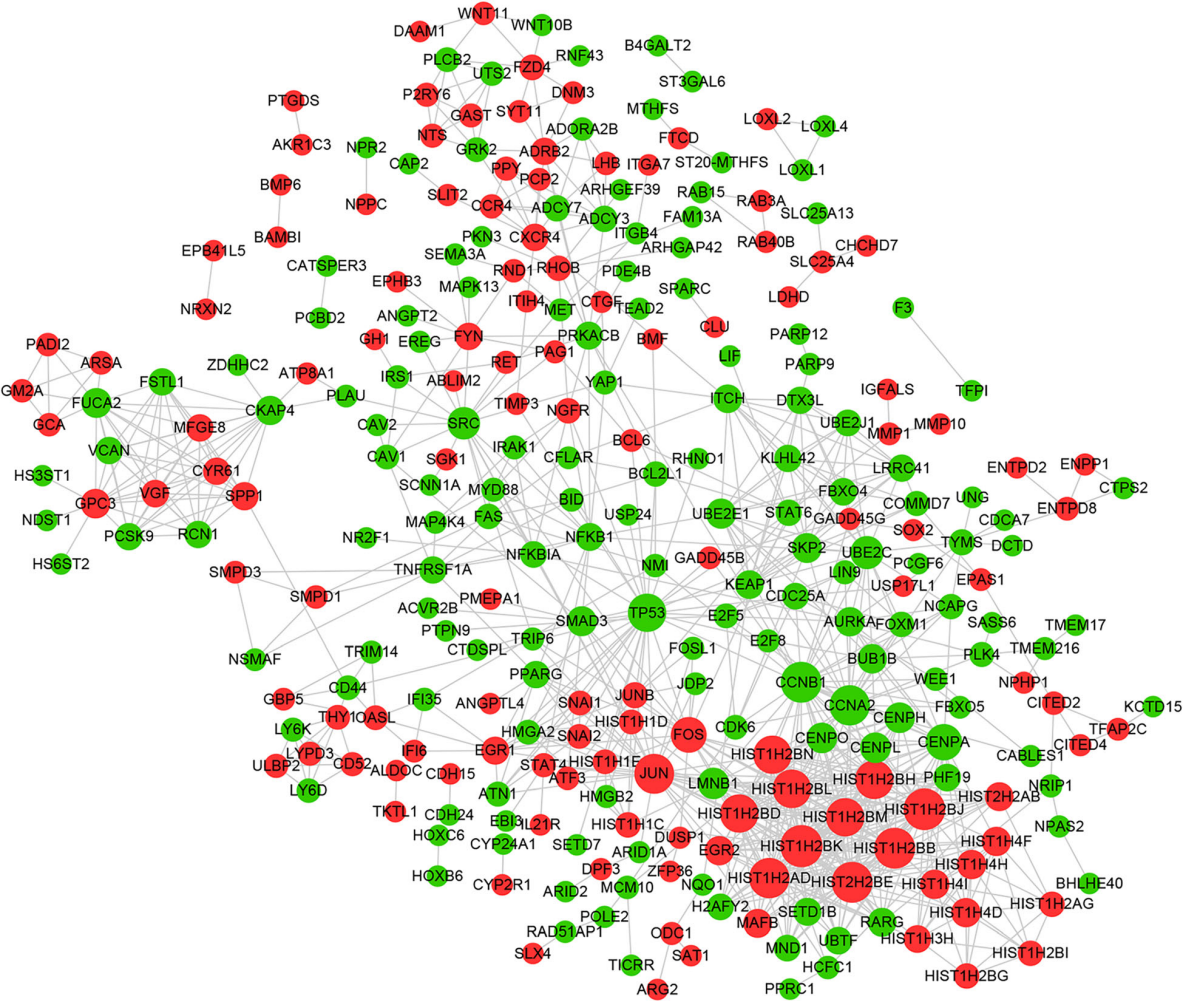
Analysis of topological properties of PPI network nodes

Node network topology analysis was carried out on the PPI network. The degree centrality (DC), betweenness centrality (BC), closeness centrality (CC) of top15 nodes are shown in this table. These proteins that appear repeatedly in the analysis results are marked in red as hub proteins

### Construction of ceRNA network

**Fig. 7:**
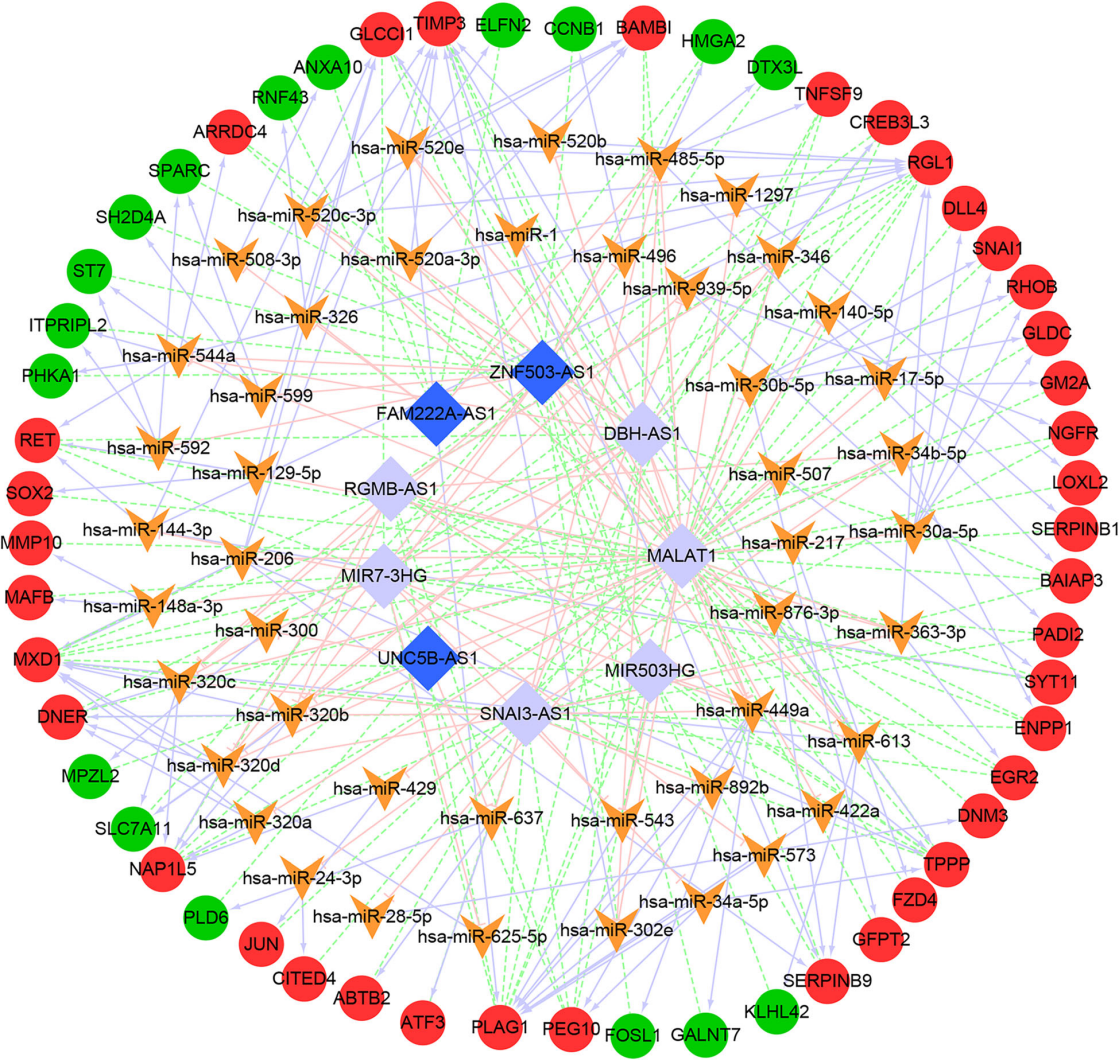
The ceRNA network. The ceRNA networks were constructed based on 686 lncRNA-miRNA-mRNA regulatory relationship pairs including 46 miRNAs, 9 lncRNAs, and 55 mRNAs by using cytoscape software. The red circle and green circle represent up-regulated and down-regulated mRNAs, respectively. The purple diamond and dark blue diamond represent up-regulated and down-regulated lncRNAs, respectively. The pink t-shaped line, light purple arrow, and green dotted line represent the regulatory relationships between lncRNA and miRNA, that between miRNA and mRNA, and the co-expression relationship between mRNAs and lncRNA, respectively

Targeted miRNA prediction based on lnCeDB was performed for the 30 lncRNA-mRNA pairs mentioned above. A total of 1025 lncRNA-miRNA pairs, including 150 miRNAs and 10 lncRNAs, were obtained. Based on miRWalk2.0 and the set threshold, 1560 miRNA-mRNA pairs, including 490 miRNAs and 203 mRNAs, were obtained. The mRNA and lncRNA regulated by the same miRNA and lncRNAs and mRNAs with a correlation coefficient of over 0.9 were screened. Finally, 686 lncRNA-miRNA-mRNA regulatory relationships, including 46 miRNAs, 9 lncRNAs, and 55 mRNAs, were obtained. A ceRNA network (Fig. 7) was constructed for these pairs using the Cytoscape software. The ceRNA network contained a total of 686 lncRNA-miRNA-mRNA regulatory relationships, 86 lncRNA-miRNA relationships, 115 miRNA-mRNA relationships, and 106 co-expressed mRNAs.

### Survival analysis based on related genes and their role in CRC prognosis

**Fig. 8:**
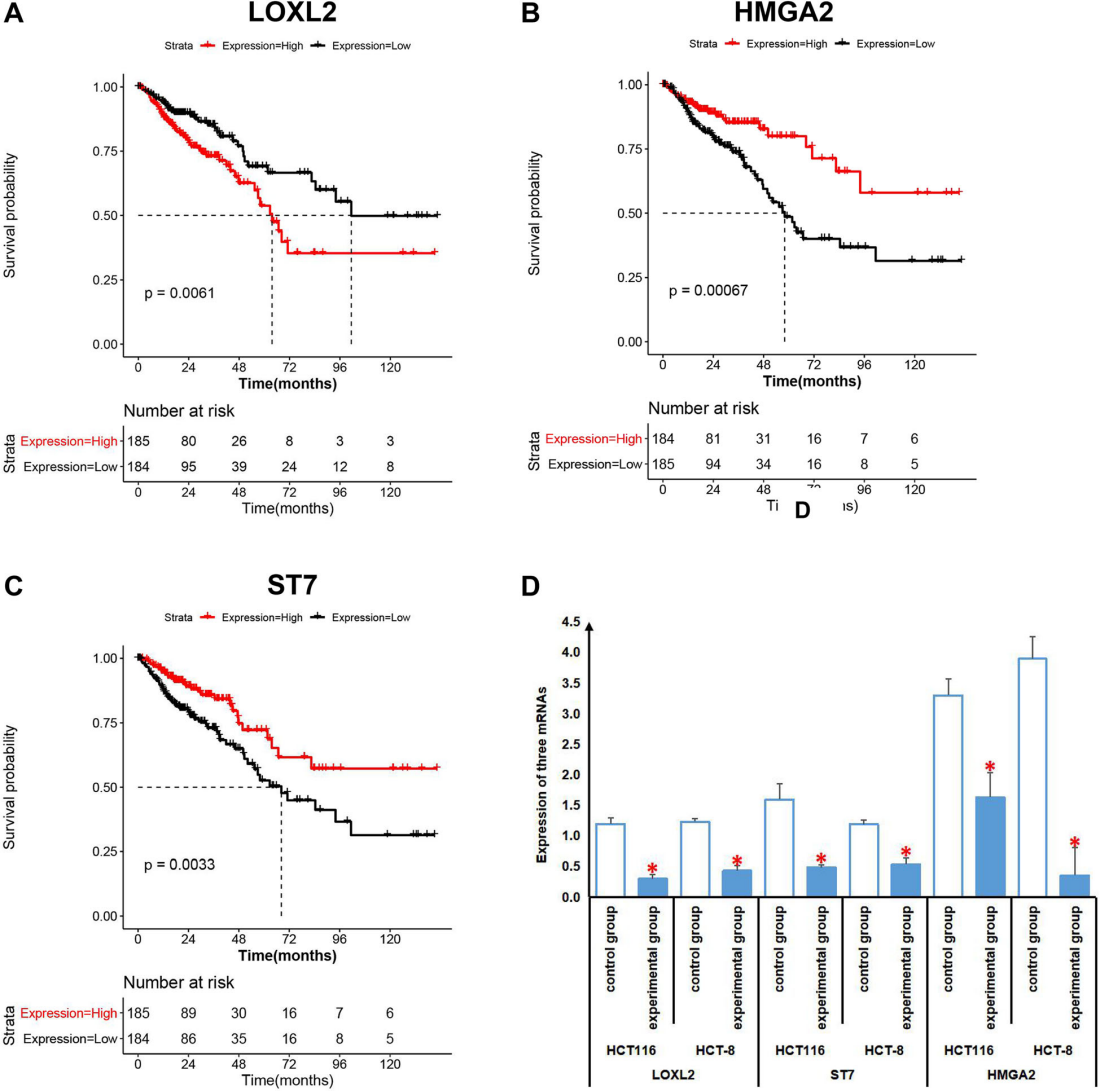
K-m survival curves. All mRNAs and lncRNAs in the ceRNA network were selected as candidate genes for survival analysis based on the gene expression value and clinical survival information of CRC from TCGA. The samples were divided into high and low risk groups according to the median value of each gene expression level. The panels **a**, **b**, and **c** respectively, show the relationship between HMGA2, LOXL2, and ST7 genes and survival time for patients with CRC. The qPCR assays were performed to determine the expression of HMGA2, LOXL2 and ST7. The panels D shows that the HCT116 and HCT8 cell lines untreated NaB were used as controls, and the HCT116 and HCT8 cell lines treated with NaB were used as experimental group. *on the bar means *P* < 0.05

All mRNAs and lncRNAs in the ceRNA network were selected as candidate genes for survival analysis. The gene expression value and clinical survival information of CRC from TCGA was used as analytical data. The samples were divided into high- and low-risk groups according to the median value of each gene expression level to conduct the survival analysis and generate the K-m survival curve. Three mRNAs, HMGA2, LOXL2, and ST7, were found to be significantly correlated with the prognosis (*p* < 0.05). As shown in Fig. 8a-c, high HMGA2, low LOXL2, and low ST7 expression individually predicted poor prognosis of CRC.

### Expression of three mRNAs associated with the prognosis

The qPCR was used for rapid and quantitative detection of HMGA2, LOXL2 and ST7.HCT116 and HCT8 cells treated with NAB were divided into experimental group and untreated group as control group. As shown in Fig. 8d, the results revealed that The expressions of HMGA2, LOXL2 and ST7 in the experimental group were significantly lower than those in the control group (*P* < 0.05).

### NaB inhibits the proliferation of CRC cells

**Fig. 9:**
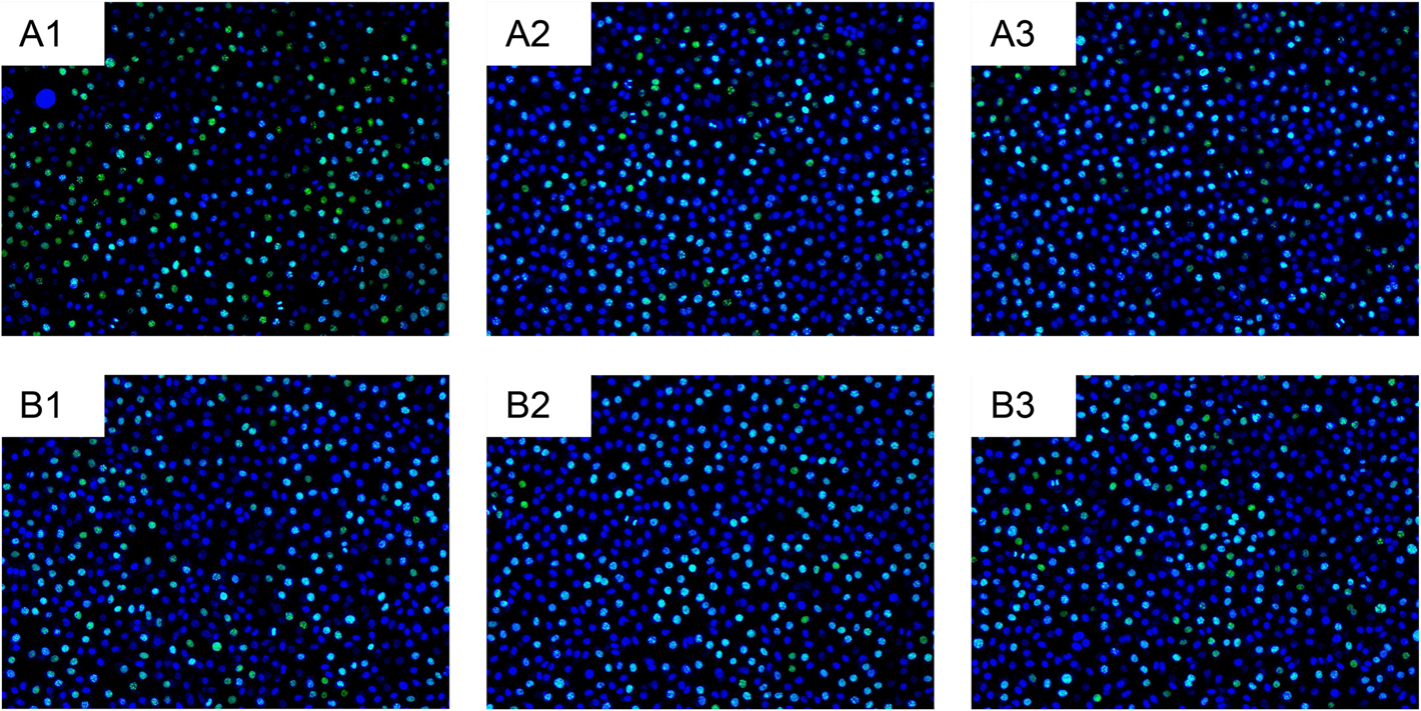
NaB inhibited the proliferation of CRC cell lines. The EDU assay was used to determine the effects of NaB on the proliferation of CRC cell lines. The panels A1, A2 and A3 represent the percentage of the S phase population in SW480 cells untreated with NaB (control group). The panels B1, B2 and B3 represent the percentage of the S phase population in SW480 cells treated with 5 mmol/L NaB for 48 h. Green circles represent proliferative cells and blue circles represent total cells

The EdU assay was applied to detect cell proliferation. In Fig. 9, The panels A1, A2 and A3 revealed the percentage of the S phase population in SW480 lines without adding NaB, which was respectively 66.2, 63.6 and 62.28%. The panels B1, B2 and B3 showed the cells treated with 5 mmol/L NaB for 48 h, as accounted for 43.32, 44.72 and 47.32%, respectively. As shown in Fig. 9, the percentage of the S phase population in SW480 cells treated with NaB was lower than that in the control group(*P* < 0.001).

## Discussion

NaB is the final product of dietary fiber decomposition by intestinal microorganisms, such as *Butyricicoccus pullicaecorum, Bifidobacterium longum, Butyrivibrio fibrisolvens, Ruminococcus bromii,* and *Lachnospiraceae*, in the colorectum [28]. Although its concentration is low in the colorectum, NaB has important biological activities in intestinal mucosal cells [29]. NaB is also involved in the occurrence and development of CRC [30]; NaB levels have been reported to be decreased in stool samples of CRC patients [31, 32]. In the present study, we investigated the biological effects of NaB on CRC and explored its underlying molecular mechanism(s) of action. We found that in vitro, NaB induced apoptosis and inhibited CRC cell proliferation, invasion, and metastasis by modulating complex molecular networks. NaB also induced G1 phase block in CRC cells. The RNA prediction and molecular network construction may provide novel directions for future research.

Although some molecular mechanistic studies have been conducted to elucidate how NaB inhibits CRC [33, 34], the molecular mechanism remains obscure because of the complexity of intracellular molecular signaling pathways. To address this, we constructed a PPI network and a ceRNA network using multiple databases. The ceRNA (competing endogenous RNA) hypothesis shows the interaction mechanisms between RNA [35]. The competition between lncRNA and microRNA leads to the change of downstream molecular expression and thus altered cell function [36, 37]. Increasing evidence in recent years has implicated that ceRNAs play important role in the development of many cancers, such as breast cancer [38], gastric cancer [39], cholangiocarcinoma [40], CRC [41] and so on. We constructed a PPI network and a ceRNA network basing on multiple databases. Our results may provide guidance and direction for further research on the molecular mechanism of NaB inhibition CRC.

The relationship between CRC prognosis and differentially expressed RNA was analyzed basing on the gene expression value and clinical survival information of CRC from TCGA. Three differentially expressed mRNAs including HMGA2, LOXL2, and ST7 were significantly correlated with the prognosis of CRC. A high expression of HMGA2 indicated a poor prognosis of CRC, and HMGA2 was significantly downregulated after NaB treatment. Low LOXL2 expression predicted the poor prognosis of CRC, and LOXL2 was significantly upregulated after NaB treatment. These two genes may be potential molecular targets of NaB in inhibiting CRC. The low ST7 expression predicted the poor prognosis of CRC. However, the ST7 gene in CRC cells is significantly downregulated after NaB treatment. It may be related to the side effects of NaB.

We found that HMGA2, LOXL2, and ST7 were significantly correlated with the prognosis of CRC. The prognostic factors are not only related to the biological function of tumor cells, but also related to recurrence and metastasis, drug sensitivity, PS score of patients, nutritional status and other factors. We will try to study the prognostic differential genes, such as HMGA2, LOXL2 and ST7 in the further studies.

There are some limitations in the present study. Although we built the network based on database analysis and RNA prediction, we did not further verify its molecular network. The critical nodes of the PPI network and ceRNA network may provide some direction for further molecular validation. In addition, although some studies have indicated that NaB is reduced in the stools from CRC patients, the interaction of numerous microorganisms and various metabolites in stools leads to a complex intestinal microecosystem. The clinical application of NaB is limited by the complexity, variability and individuality of intestinal microecosystem.

## Conclusions

We investigated the effects of NaB on CRC cells. In addition, we explored its molecular pathways basing on database analysis and RNA prediction. NaB induces the apoptosis and inhibition of CRC cell proliferation, invasion, and metastasis by modulating complex molecular networks. This will provide a theoretical basis for NaB as an anticancer agent.

## Data Availability

The datasets generated during the current study are not publicly available but de-identified and anonymized information is potentially available on reasonable request.

## Abbreviations

CRC: Colorectal Cancer
NaB: Sodium Butyrate
CCK-8: Cell Counting Kit-8
RNA: Ribonucleic Acid
lncRNA: Long Non-coding Ribonucleic Acid
mRNA: Messenger Ribonucleic Acid
PPI: Protein-Protein Interaction
miRNA: Micro Ribonucleic Acid
ceRNA: Competing Endogenous Ribonucleic Acid
HMGA2: High Mobility Group AT-hook 2
LOXL2: Lysyl Oxidase-like 2
HDAC: Histone Deacetylase
PBS: Phosphate-Buffered Saline
FACS: Fluorescence-Activated Cell Sorting
GEO: Gene Expression Omnibus
KEGG: Kyoto Encyclopedia of Genes and Genomes
COADREAD: Colorectal Adenocarcinoma
DC: Degree Centrality
BC: Betweenness Centrality
CC: Closeness Centrality
OS: Overall Survival
TCGA: The Cancer Genome Atlas
M: Kaplan-Meier
IC50: Half Maximal Inhibitory Concentration
BP: Biological Processes
TNF: Tumor Necrosis Factor
DC: Degree Centrality

## References

[1] Barresi V, Reggiani Bonetti L, Ieni A, et al. Poorly differentiated clusters (PDC): clinical impact in colorectal cancer[J]. Clin Color Cancer. 2017;16(1):9-15. 10.1016/j.clcc.2016.06.002 27444718

[2] Trowbridge B, Burt RW (1993). Colorectal cancer screening.[J]. J Community Health.

[3] Golovko D, Kedrin D, Yilmaz ÖH, Roper J (2015). Colorectal cancer models for novel drug discovery [J]. Expert Opin Drug Discov.

[4] Alessia B, Elena DM, Erika C (2017). Pharmacogenomics of targeted agents for personalization of colorectal Cancer treatment [J]. Int J Mol Sci.

[5] Coppedè F, Lopomo A, Spisni R, Migliore L (2014). Genetic and epigenetic biomarkers for diagnosis, prognosis and treatment of colorectal cancer [J]. World J Gastroenterol.

[6] Joranger P, Nesbakken A, Hoff G (2015). Modeling and validating the cost and clinical pathway of colorectal Cancer [J]. Med Decis Mak.

[7] Kilner J, Corfe BM, Mcauley MT (2016). A deterministic oscillatory model of microtubule growth and shrinkage for differential actions of short chain fatty acids [J]. Mol BioSyst.

[8] Wei W, Zhibin X, Wenyi A (2018). Dietary sodium butyrate improves intestinal development and function by modulating the microbial community in broilers [J]. PLoS One.

[9] Sun J, Kato I (2016). Gut microbiota, inflammation and colorectal cancer [J]. Annu Rev Microbiol.

[10] Geirnaert A, Calatayud M, Grootaert C (2017). Butyrate-producing bacteria supplemented in vitro to Crohn's disease patient microbiota increased butyrate production and enhanced intestinal epithelial barrier integrity [J]. Sci Rep.

[11] Saldanha SN, Kala R, Tollefsbol TO (2014). Molecular mechanisms for inhibition of colon cancer cells by combined epigenetic-modulating epigallocatechin gallate and sodium butyrate [J]. Exp Cell Res.

[12] Wu JT, Archer SY, Hinnebusch B (2001). Transient vs. prolonged histone hyperacetylation: effects on colon cancer cell growth, differentiation, and apoptosis [J]. Am J Physiol Gastrointest Liver Physiol.

[13] Gonçalves P, Martel F (2013). Butyrate and colorectal Cancer: the role of butyrate transport [J]. Curr Drug Metab.

[14] Barrett T, Suzek TO, Troup DB (2005). NCBI GEO: mining millions of expression profiles—database and tools [J]. Nucleic Acids Res.

[15] Gautier L, Cope L, Bolstad BM (2004). Affy--analysis of Affymetrix GeneChip data at the probe level [J]. Bioinformatics.

[16] Harrow J, Frankish A, Gonzalez JM (2012). GENCODE: the reference human genome annotation for the ENCODE project [J]. Genome Res.

[17] Jiang H, Wong WH (2008). SeqMap: mapping massive amount of oligonucleotides to the genome [J]. Bioinformatics.

[18] Smyth GK. limma: Linear Models for Microarray Data[J]. Bioinformatics and Computational Biology Solutions Using R and Bioconductor. 2005;397-420. 10.1007/0-387-29362-0_23.

[19] Thissen D, Steinberg L, Kuang D (2002). Quick and easy implementation of the Benjamini-Hochberg procedure for controlling the false positive rate in multiple comparisons [J]. J Educ Behav Stat.

[20] Yu G, Wang LG, Han Y (2012). clusterProfiler: an R package for comparing biological themes among gene clusters [J]. OMICS.

[21] Ashburner M, Ball CA, Blake JA (2000). Gene ontology: tool for the unification of biology [J]. Gene.

[22] Kanehisa M, Goto S, Kawashima S (2000). KEGG: Kyoto encyclopaedia of genes and genomes [J]. Nucleic Acids Res.

[23] Franceschini A, Szklarczyk D, Frankild S (2012). STRING V9.1: Protein-Protein Interaction Networks, with Increased Coverage and Integration [J]. Nucleic Acids Res.

[24] Shannon P (2003). Cytoscape: a software environment for integrated models of biomolecular interaction networks [J]. Genome Res.

[25] Tang Y, Li M, Wang J (2015). CytoNCA: a cytoscape plugin for centrality analysis and evaluation of protein interaction networks [J]. Biosystems.

[26] Shaoli D, Suman G, Rituparno S (2014). lnCeDB: Database of Human Long Noncoding RNA Acting as Competing Endogenous RNA [J]. PLoS ONE.

[27] Dweep H, Gretz N (2015). miRWalk2.0: a comprehensive atlas of microRNA-target interactions [J]. Nat Methods.

[28] Faraz B, Phillip E, Nailliw P (2018). Dietary Fiber treatment corrects the composition of gut microbiota, promotes SCFA production, and suppresses Colon carcinogenesis [J]. Genes.

[29] O’Keefe SJD (2016). Diet, microorganisms and their metabolites, and colon cancer [J]. Nat Rev Gastroenterol Hepatol.

[30] Shuwen H, Miao D, Quan Q (2019). Protective effect of the “food-microorganism-SCFAs” axis on colorectal cancer: from basic research to practical application [J]. J Cancer Res Clin Oncol.

[31] Chen HM, Yu YN, Wang JL (2013). Decreased dietary fiber intake and structural alteration of gut microbiota in patients with advanced colorectal adenoma [J]. Am J Clin Nutr.

[32] Hamer H (2008). Review article: the role of butyrate on colonic function [J]. Aliment Pyarmacol Ther.

[33] Pryde SE, Duncan SH, Hold GL (2002). The microbiology of butyrate formation in the human colon [J]. FEMS Microbiol Letters.

[34] Xu Z, Tao J, Chen P (2018). Sodium Butyrate Inhibits Colorectal Cancer Cell Migration by downregulating Bmi-1 through enhanced miR-200c expression [J]. Mol Nutr Food Res.

[35] Shuwen H, Qing Z, Yan Z (2018). Competitive endogenous RNA in colorectal cancer: A systematic review [J]. Gene.

[36] Ling F, Du WW, Xiangling Y (2013). Versican 3′-untranslated region (3′-UTR) functions as a ceRNA in inducing the development of hepatocellular carcinoma by regulating miRNA activity.[J]. FASEB J.

[37] Barbagallo C, Brex D, Caponnetto A (2018). LncRNA UCA1, Upregulated in CRC Biopsies and Downregulated in Serum Exosomes, Controls mRNA Expression by RNA-RNA Interactions [J]. Mol Ther Nucleic Acids.

[38] Abdollahzadeh R, Daraei A, Mansoori Y (2019). Competing endogenous RNA (ceRNA) cross talk and language in ceRNA regulatory networks: A new look at hallmarks of breast cancer [J]. J Cellular Physiol.

[39] Arun K, Arunkumar G, Bennet D, et al. Comprehensive analysis of aberrantly expressed lncRNAs and construction of ceRNA network in gastric cancer[J]. Oncotarget. 2018;9(26):18386-99. 10.18632/oncotarget.24841 PMC5915079 29719612

[40] Li G, Liu T, Zhang B, et al. Genome-wide identification of a competing endogenous RNA network in cholangiocarcinoma[J]. J Cell Biochem. 2019;120(11):18995-9003. 10.1002/jcb.29222 PMC6771781 31270845

[41] He F, Song Z, Chen H, et al. Long noncoding RNA PVT1-214 promotes proliferation and invasion of colorectal cancer by stabilizing Lin28 and interacting with miR-128.[J]. Oncogene. 2018;38(2):164-79. 10.1038/s41388-018-0432-8 PMC6329639 30076414

